# Impact of inactivated SARS-CoV-2 vaccination on embryo ploidy: a retrospective cohort study of 133 PGT-A cycles in China

**DOI:** 10.1186/s40659-022-00395-1

**Published:** 2022-08-12

**Authors:** Jialyu Huang, Leizhen Xia, Lifeng Tian, Hancheng Fan, Dingfei Xu, Xiaoyan Ai, Xingwu Wu, Jia Chen, Genbao Xing, Lingling Huang, Huijun Zuo, Jia Chen, Mengxi Li, Ke Zhang, Peipei Liu, Jiaying Lin, Qiongfang Wu

**Affiliations:** 1 Center for Reproductive Medicine, Jiangxi Maternal and Child Health Hospital, 318 Bayi Avenue, Nanchang, 330006 China; 2 School of Basic Medicine, Nanchang University, Nanchang, China; 3 Department of Gynecology, Jiangxi Maternal and Child Health Hospital, Nanchang, China; 4 Department of Assisted Reproduction, Shanghai Ninth People’s Hospital, Shanghai Jiao Tong University School of Medicine, 639 Zhizaoju Rd, Shanghai, 200011 China

**Keywords:** COVID-19, SARS-CoV-2, Inactivated vaccine, Embryo ploidy, In vitro fertilization, Preimplantation genetic testing

## Abstract

**Background:**

Unsubstantiated concerns have been raised on the potential correlation between severe acute respiratory syndrome coronavirus 2 (SARS-CoV-2) vaccination and infertility, leading to vaccine hesitancy in reproductive-aged population. Herein, we aim to evaluate the impact of inactivated SARS-CoV-2 vaccination on embryo ploidy, which is a critical indicator for embryo quality and pregnancy chance.

**Methods:**

This was a retrospective cohort study of 133 patients who underwent preimplantation genetic testing for aneuploidy (PGT-A) cycles with next-generation sequencing technology from June 1st 2021 to March 17th 2022 at a tertiary-care medical center in China. Women fully vaccinated with two doses of Sinopharm or Sinovac inactivated vaccines (*n* = 66) were compared with unvaccinated women (*n* = 67). The primary outcome was the euploidy rate per cycle. Multivariate linear and logistic regression analyses were performed to adjust for potential confounders.

**Results:**

The euploidy rate was similar between vaccinated and unvaccinated groups (23.2 ± 24.6% *vs.* 22.6 ± 25.9%, *P* = 0.768), with an adjusted β of 0.01 (95% confidence interval [CI]: -0.08–0.10). After frozen-thawed single euploid blastocyst transfer, the two groups were also comparable in clinical pregnancy rate (75.0% *vs.* 60.0%, *P* = 0.289), with an adjusted odds ratio of 6.21 (95% CI: 0.76–50.88). No significant associations were observed between vaccination and cycle characteristics or other laboratory and pregnancy outcomes.

**Conclusions:**

Inactivated SARS-CoV-2 vaccination had no detrimental impact on embryo ploidy during in vitro fertilization treatment. Our finding provides further reassurance for vaccinated women who are planning to conceive. Future prospective cohort studies with larger datasets and longer follow-up are needed to confirm the conclusion.

## Background

Severe acute respiratory syndrome coronavirus 2 (SARS-CoV-2) has caused nearly 500 million cases of coronavirus disease 19 (COVID-19), with more than six million deaths attributed to it [1]. To reduce morbidity and mortality, nationwide vaccination programs have been promoted around the world. As of 4 April 2022, a total of 11.2 billion vaccine doses have been administered globally, including 3.2 billion doses in Chinese mainland [1, 2]. Compared with messenger ribonucleic acid (mRNA) and viral-vector vaccines in other countries, inactivated SARS-CoV-2 vaccines are predominantly used in China for its mature production technology, easy storage requirement and comparably high efficacy [3].

Despite reassuring safety profiles reported in clinical trials [3–5], vaccine hesitancy in reproductive-aged population has been heightened due to the spread of misinformation on social media platforms claiming a negative effect of COVID-19 vaccine on fertility [6, 7]. In the 48 days following Emergency Use Authorization, internet search volume in Google related to COVID‐19 vaccine and fertility significantly increased by 207.56% to 2943.7% [8], implying the need of scientific evidences to address public concern.

Several studies to date have found no association between mRNA COVID-19 vaccine and sperm parameters [9–12], follicular function [13–17] as well as embryo development [14, 15]. With a specific focus on in vitro fertilization (IVF) cycles using preimplantation genetic testing for aneuploidy (PGT-A), one study further demonstrated similar euploidy rate after mRNA COVID-19 vaccination on adjusted analysis [17]. Nonetheless, it remains unclear whether inactivated SARS-CoV-2 vaccine results in any measurable influence on the status of euploidy, which is a critical indicator for both embryo quality and pregnancy chance [18].

The objective of the present work was to evaluate the effect of inactivated SARS-CoV-2 vaccination on blastocyst ploidy in patients undergoing PGT-A cycles.

## Materials and methods

### Study design and population

This retrospective cohort study was conducted at the Center for Reproductive Medicine, Jiangxi Maternal and Child Health Hospital. From June 1st 2021 to March 17th 2022, all infertile patients who underwent oocyte retrieval cycles with PGT-A were reviewed for eligibility and followed-up for pregnancy outcomes until June 10th 2022. The indications for PGT-A included advanced maternal age (≥ 38 years old), recurrent pregnancy loss, recurrent implantation failure and others, such as severe male factor and previous conception with aneuploidy.

Routinely, screening for COVID-19 was carried out for each patient before treatment initiation, including symptom questionnaire, temperature measurement, chest X-ray film, polymerase chain reaction test for SARS-CoV-2 RNA, as well as survey for past infection history and vaccination status. For vaccinated patients, we further collected information on vaccine type, manufacturer, dose, date and location, which were ascertained via official records in national (e.g., Alipay and WeChat) as well as local (e.g., Ganfutong and Changtongma) mobile applets. Patients were classified into the study group if they received two doses of Sinopharm or Sinovac COVID-19 vaccines with an interval of three weeks or longer, while the control group consisted of unvaccinated patients. Cycles involving one of the following criteria were excluded from the study: 1) history of SARS-CoV-2 infection; 2) partial vaccination without the second dose; 3) administration of other types of COVID-19 vaccines, namely viral-vector vaccine (CanSino) and protein-based vaccine (ZF2001) in China [19, 20]; 4) cycles using donor oocyte/sperm for fertilization or cleavage-stage embryo for biopsy; 5) parental abnormalities in chromosomal structures or diagnosis of monogenic diseases; and 6) missing data in the electronic medical records. To avoid confounding, only the first cycle was retained for analysis in cases of two or more PGT-A cycles from the same patient. No loss to follow-up was found during the timeframe. This study was approved by the Reproductive Medicine Ethics Committee of Jiangxi Maternal and Child Health Hospital (No. 2022–01), with informed contents obtained from patients for de-identified data use in scientific research.

### IVF procedures

Controlled ovarian stimulation was performed as described elsewhere [21, 22], including early-follicular phase long-acting gonadotropin-releasing hormone (GnRH) agonist long protocol, mid-luteal phase short-acting GnRH agonist long protocol, flexible GnRH antagonist protocol and progestin-primed ovarian stimulation protocol. Follicular growth was monitored by transvaginal ultrasonography and serum sex hormone measurement. Once the leading follicle reached 20 mm or two follicles reached 18 mm in diameter, recombinant human chorionic gonadotropin (hCG; Ovidrel, Merck Serono, Switzerland), or a combination of urinary hCG (Lizhu Pharmaceuticals, China) and triptorelin acetate (Decapeptyl, Ferring Pharmaceuticals, Germany) was administrated to induce final oocyte maturation. Oocyte retrieval was performed 36–38 h after triggering.

To avoid contamination with sperm deoxyribonucleic acid (DNA), all metaphase II (MII) oocytes were inseminated via intracytoplasmic sperm injection after denudation, followed by pronuclei (PN) assessment 16–18 h later. Embryos were cultured to the blastocyst stage in sequential G1-plus/G2-plus medium (Vitrolife, Sweden) at 37 °C under a 6.5% CO_2_ and 5% O_2_ atmosphere. On day 5 or 6, morphological scoring was performed based on the Gardner and Schoolcraft’s system, including expansion degree, inner cell mass and trophectoderm development [23]. Blastocyst with a grading over 4CC was considered as usable for further biopsy.

After embryo stabilization by holding pipette, approximately 3–7 trophectoderm cells were aspirated using a biopsy pipette (30-mm internal diameter) and dissected with laser pulsation (Saturn 5 Active, Research Instruments, UK). The isolated cells were then subjected to whole genome amplification with commercial kits (Basecare Medical Device, China). The amplified products were fragmented to an average size of 170 bp and end-ligated using sequencing adaptors with identifiable barcodes for the Ion Proton platform. Next-generation sequencing (NGS) was performed in DA8600 (Daan Gene, China) and the copy number variation of each embryo was analyzed. Results of PGT-A were reported as euploid, aneuploid and mosaic in occurrence of < 30%, > 70% and 30–70% of abnormal cells, respectively. Biopsied blastocysts were all cryopreserved by vitrification.

For frozen-thawed embryo transfer (FET), hormone replacement therapy was used for endometrial preparation as previously described [24]. Oral estradiol valerate (4–6 mg/d; Progynova, Bayer, Germany) was commenced from the 3rd day of menstruation. After 7 days, ultrasound examination was carried out to ensure the absence of dominant follicle and the estradiol dose was adjusted according to endometrial thickness (EMT). When an EMT of 7 mm was achieved with a minimal treatment duration for 12 days, daily progesterone was given through intramuscular (80 mg/d; Xianju Pharma, China) or oral (20 mg/d; Duphaston, Abbott Biologicals, USA) and vaginal (90 mg/d; Crinone, Merck Serono, Switzerland) routes. FET was scheduled at 5 days following endometrial transformation and single euploid blastocyst was transferred in all cycles.

### Outcome measures and definitions

The primary outcome of the study was euploidy rate per ovarian stimulation cycle, defined as the ratio of the number of euploid embryos to the number of biopsied embryos. Other laboratory outcomes included oocyte retrieval rate (retrieved oocytes out of ≥ 14 mm follicles on trigger day), mature oocyte rate (MII oocytes out of retrieved oocytes), normal fertilization rate (2PN oocytes out of MII oocytes), cleavage rate (day 3 embryos developed from 2PN oocytes out of 2PN oocytes), blastulation rate (blastocysts out of day 3 embryos for extended culture), aneuploidy rate (aneuploid embryos out of biopsied embryos), and mosaicism rate (mosaic embryos out of biopsied embryos).

We also evaluated pregnancy outcomes in subsequent euploid FET cycles. Biochemical pregnancy was defined as positive hCG test with serum concentration ≥ 5 mIU/mL after blastocyst transfer for 10–12 days. Clinical pregnancy was identified as the presence of gestational sac with fetal heart beat at one month following transfer. Biochemical pregnancy loss rate was calculated as loss of positive hCG before detection of clinical pregnancy out of all biochemical pregnancies.

### Statistical analysis

For continuous variables, data were expressed as means with standard deviations and assessed for distribution using the Shapiro–Wilk test. Data complying with normality were compared by Student's t-test, while Mann–Whitney U-test was used for non-normal data. For categorical variables, data were presented as numbers with percentages, and between-group differences were compared by Pearson’s Chi-square test or Fisher’s exact test as appropriate.

In order to evaluate the independent effect of inactivated SARS-CoV-2 vaccination, multivariate regression analyses were further performed to control for potential confounders determined a priori. For embryo ploidy outcomes, β coefficients and 95% confidence intervals (CIs) were estimated from generalized linear regression models. Adjusted covariates included parental age and body mass index (BMI), male vaccination status, infertility type, duration and diseases, anti-müllerian hormone (AMH) level, antral follicle count (AFC), ovarian stimulation protocol, and indication for PGT-A. For FET pregnancy outcomes, odds ratios (ORs) and 95% CIs were calculated from Firth's penalized likelihood logistic regression models. Covariates included in the adjusted analysis were female age and BMI, male vaccination status, infertility type, duration and diseases, AMH, AFC, EMT, embryo developmental stage, expansion grade, inner cell mass grade, and trophectoderm grade. SPSS version 20.0 (SPSS Inc., USA) and SAS version 9.4 (SAS Institute, USA) were used for all data analyses. All tests were two-tailed and *P* < 0.05 was considered to be statistically significant.

## Results

In total, 133 patients fulfilled the eligibility criteria, including 66 fully vaccinated and 67 unvaccinated patients. For vaccinated women, the time interval from second-dose completion to IVF cycle initiation ranged between 7 and 317 days (mean: 126.5 ± 64.0 days).

Table 1 displays the demographic features according to female vaccination status. The control group had a significantly longer duration of infertility (3.7 ± 3.3 *vs.* 2.6 ± 2.5 years, *P* = 0.042) and higher proportion of unvaccinated males (68.7% *vs.* 21.2%, *P* < 0.001) compared with the vaccinated group. No significant differences were observed in parental age, parental BMI, infertility type and diseases, ovarian reserve, semen quality, stimulation protocol, and PGT-A indications. All patients were of Han ethnicity, and no couples reported a prior SARS-CoV-2 infection history.

**Table 1:** Demographic features of the study groups

	Vaccinated (*n* = 66)	Unvaccinated (*n* = 67)	*P*-value
Female age (years)	37.7 ± 5.2	37.7 ± 4.5	0.867
Female BMI (kg/m^2^)	21.9 ± 2.1	22.6 ± 2.7	0.127
Infertility duration (years)	2.6 ± 2.5	3.7 ± 3.3	0.042
Infertility type, *n* (%)			0.563
Primary	5 (7.6)	7 (10.5)	
Secondary	61 (92.4)	60 (89.6)	
Infertility diseases			
Tubal factor, *n* (%)	27 (40.9)	25 (37.3)	0.671
Male factor, *n* (%)	13 (19.7)	9 (13.4)	0.331
Ovulatory dysfunction, *n* (%)	4 (6.1)	4 (6.0)	1.000
Diminished ovarian reserve, *n* (%)	20 (30.3)	27 (40.3)	0.228
Endometriosis, *n* (%)	2 (3.0)	4 (6.0)	0.414
Uterine factor, *n* (%)	7 (10.6)	9 (13.4)	0.616
AMH (ng/mL)	2.8 ± 1.8	3.1 ± 2.5	0.698
Antral follicle count	11.4 ± 5.1	10.6 ± 5.0	0.604
Male age (years)	39.8 ± 6.6	38.7 ± 6.0	0.394
Male BMI (kg/m^2^)	24.6 ± 5.1	24.3 ± 5.0	0.508
Male vaccination status, *n* (%)			< 0.001
Unvaccinated	14 (21.2)	46 (68.7)	
Partially vaccinated	3 (4.6)	4 (6.0)	
Fully vaccinated	49 (74.2)	17 (25.4)	
Total semen volume (mL)	2.7 ± 1.1	3.0 ± 1.0	0.074
Sperm concentration (10^6^/mL)	73 ± 48.8	67.4 ± 50.2	0.366
Progressive sperm motility (%)	36.9 ± 14.8	33 ± 15.0	0.150
Normal sperm morphology (%)	5.4 ± 1.4	5.5 ± 1.5	0.882
Ovarian stimulation protocol, *n* (%)			0.997
GnRH antagonist	22 (33.3)	22 (32.8)	
MLSL	26 (39.4)	26 (38.8)	
EFLL	8 (12.1)	9 (13.4)	
PPOS	10 (15.2)	10 (14.9)	
Indication for PGT-A, *n* (%)			0.919
Advanced maternal age	33 (50.0)	31 (46.3)	
Recurrent pregnancy loss	24 (36.4)	24 (35.8)	
Recurrent implantation failure	5 (7.6)	7 (10.5)	
Other	4 (6.1)	5 (7.5)	

Data are presented as mean ± standard deviation or number (percentage)

*BMI* body mass index, *AMH* anti-müllerian hormone, *GnRH* gonadotropin-releasing hormone, *MLSL* mid-luteal phase short-acting GnRH agonist long protocol, *EFLL* early-follicular phase long-acting GnRH agonist long protocol, *PPOS* progestin-primed ovarian stimulation protocol, *PGT-A* preimplantation genetic testing for aneuploidy

Cycle characteristics and laboratory outcomes are presented in Table 2. The primary outcome of euploidy rate was similar between vaccinated and unvaccinated women (23.2 ± 24.6% *vs.* 22.6 ± 25.9%, *P* = 0.768), in accordance with the similar number of euploid embryos (1.0 ± 1.3 *vs.* 1.0 ± 1.3, *P* = 0.954). Consistently, there were no significant differences in the number and proportion of both aneuploid and mosaic embryos. Other laboratory outcomes, including mature oocyte ratio, normal fertilization rate and blastulation rate, were also comparable between the two groups. No differences were seen in cycle characteristics with regard to length of stimulation, total gonadotropin dose, sex hormone profile and number of ≥ 14 mm follicles on trigger day.

**Table 2:** Cycle characteristics and laboratory outcomes grouped by the vaccination status

	Vaccinated (*n* = 66)	Unvaccinated (*n* = 67)	*P*-value
Stimulation duration (days)	9.2 ± 1.3	9.5 ± 1.7	0.321
Total gonadotropin dose (IU)	2105.3 ± 476.7	2167.5 ± 563.9	0.764
LH level on trigger day (mIU/mL)	3.1 ± 2.4	2.7 ± 1.7	0.521
E_2_ level on trigger day (pg/mL)	2026.4 ± 1328.2	2005.7 ± 1150.2	0.721
P level on trigger day (ng/mL)	0.6 ± 0.7	0.5 ± 0.4	0.693
No. of ≥ 14 mm follicles on trigger day	8.2 ± 4.5	8.9 ± 4.1	0.269
No. of oocytes retrieved	12.2 ± 7.5	12.2 ± 6.9	0.948
No. of MII oocytes	9.3 ± 5.7	9.5 ± 5.5	0.878
No. of 2PN oocytes	8.2 ± 4.9	8.2 ± 5.2	0.923
No. of cleaved embryos	8.0 ± 4.9	8.0 ± 5.1	0.912
No. of usable blastocysts	3.8 ± 2.6	4.0 ± 3.4	0.841
No. of biopsied blastocysts	3.4 ± 2.0	3.7 ± 3.0	0.805
No. of aneuploid embryos	1.7 ± 1.4	1.6 ± 1.5	0.749
No. of mosaic embryos	0.7 ± 1.0	1.0 ± 1.3	0.222
No. of euploid embryos	1.0 ± 1.3	1.0 ± 1.3	0.954
Oocyte retrieval rate (%)	156.0 ± 70.6	135.5 ± 38.4	0.186
Mature oocyte rate (%)	79.3 ± 13.5	78.3 ± 13.8	0.883
Normal fertilization rate (%)	88.0 ± 14.7	86.3 ± 14.4	0.357
Cleavage rate (%)	97.9 ± 4.9	96.9 ± 9.8	0.886
Blastulation rate (%)	56.8 ± 20.9	58.2 ± 21.2	0.549
Aneuploidy rate (%)	57.4 ± 37.8	53.6 ± 35.3	0.563
Mosaicism rate (%)	19.4 ± 25.9	23.8 ± 28.5	0.406
Euploidy rate (%)	23.2 ± 24.6	22.6 ± 25.9	0.768

Data are presented as mean ± standard deviation

*LH* luteinizing hormone, *E*_*2*_ estradiol, *P* progesterone, *MII* metaphase II, *2PN* two pronuclei

Table 3 demonstrates the relationship between vaccination status and embryo ploidy outcomes on crude and adjusted analysis. After controlling for potential confounding factors, inactivated SARS-CoV-2 vaccination remained unassociated with euploidy rate (adjusted β = 0.01, 95% CI: − 0.08–0.10) and number of euploid embryos (adjusted β = 0.02, 95% CI: − 0.41–0.44). Similarly, we did not detect significantly increased risks in the number of aneuploid embryos (adjusted β = − 0.10, 95% CI: − 0.63–0.42), number of mosaic embryos (adjusted β = − 0.67, 95% CI: − 1.41–0.07), aneuploidy rate (adjusted β = 0.03, 95% CI: − 0.09–0.15), and mosaicism rate (adjusted β = − 0.04, 95% CI: − 0.14–0.06).

**Table 3:** Association between vaccination and embryo ploidy on crude and adjusted analysis

	Crude β (95% CI)	Adjusted β (95% CI)^*^	Adjusted *P*-value*
No. of aneuploid embryos	0.06 (-0.42–0.53)	-0.10 (-0.63–0.42)	0.696
No. of mosaic embryos	-0.30 (-0.70–0.09)	-0.67 (-1.41–0.07)	0.076
No. of euploid embryos	0.03 (-0.41–0.47)	0.02 (-0.41–0.44)	0.942
Aneuploidy rate (%)	0.04 (-0.09–0.16)	0.03 (-0.09–0.15)	0.629
Mosaicism rate (%)	-0.04 (-0.14–0.05)	-0.04 (-0.14–0.06)	0.445
Euploidy rate (%)	0.01 (-0.08–0.09)	0.01 (-0.08–0.10)	0.850

CI, confidence interval

^*^Adjusted for parental age and body mass index, male vaccination status, infertility type, duration and diseases, anti-müllerian hormone, antral follicle count, ovarian stimulation protocol, and indication for PGT-A

Pregnancy outcomes of subsequent single euploid FET cycles are shown in Table 4. A total of 45 patients completed 20 transfers in the vaccinated group and 25 transfers in the unvaccinated group. All baseline characteristics were comparably distributed, including maternal age and BMI, infertility type, duration and diseases, AMH, AFC, EMT, as well as male vaccination status, embryo stage, and morphological grade. Clinical pregnancy rate was 75.0% and 60.0% in vaccinated and unvaccinated patients respectively (*P* = 0.289), with an adjusted OR of 6.21 (95% CI: 0.76–50.88) (Table 5). Similarly, the rates of biochemical pregnancy (80.0% *vs.* 76.0%, *P* = 0.748) and biochemical pregnancy loss (6.3% *vs.* 21.1%, *P* = 0.213) did not differ significantly between the two groups, which remained consistent in adjusted analysis.

**Table 4:** Pregnancy outcomes following single euploid blastocyst transfer

	Vaccinated (*n* = 20)	Unvaccinated (*n* = 25)	*P*-value
Female age (years)	36.1 ± 4.7	35.9 ± 4.6	0.721
Female BMI (kg/m^2^)	21.7 ± 1.9	22.3 ± 2.6	0.268
Infertility duration (years)	2.1 ± 1.7	3.5 ± 3.2	0.177
Infertility type, *n* (%)			0.617
Primary	1 (5.0)	3 (12.0)	
Secondary	19 (95.0)	22 (88.0)	
Infertility diseases			
Tubal factor, *n* (%)	7 (35.0)	7 (28.0)	0.614
Male factor, *n* (%)	4 (20.0)	2 (8.0)	0.383
Ovulatory dysfunction, *n* (%)	1 (5.0)	0 (0)	0.444
Diminished ovarian reserve, *n* (%)	3 (15.0)	4 (16.0)	1.000
Endometriosis, *n* (%)	0 (0)	1 (4.0)	1.000
Uterine factor, *n* (%)	1 (5.0)	2 (8.0)	1.000
AMH (ng/mL)	3.5 ± 2.1	3.4 ± 2.4	0.679
Antral follicle count	13.5 ± 5.8	12.8 ± 4.5	0.819
Endometrial thickness (mm)	9.9 ± 2.4	9.0 ± 1.4	0.249
Male vaccination status, *n* (%)			0.083
Unvaccinated	7 (35.0)	16 (64.0)	
Partially vaccinated	1 (5.0)	2 (8.0)	
Fully vaccinated	12 (60.0)	7 (28.0)	
Embryo developmental stage, *n* (%)			0.945
Day 5	13 (65.0)	16 (64.0)	
Day 6	7 (35.0)	9 (36.0)	
Expansion grade, *n* (%)			0.958
4	17 (85.0)	22 (88.0)	
5	2 (10.0)	2 (8.0)	
6	1 (5.0)	1 (4.0)	
Inner cell mass grade, *n* (%)			0.617
A	0 (0)	1 (4.0)	
B	19 (95.0)	21 (84.0)	
C	1 (5.0)	3 (12.0)	
Trophectoderm grade, *n* (%)			0.633
A	0 (0)	1 (4.0)	
B	12 (60.0)	17 (68.0)	
C	8 (40.0)	7 (28.0)	
Biochemical pregnancy, *n* (%)	16 (80.0)	19 (76.0)	0.748
Biochemical pregnancy loss, *n/N* (%)	1 (6.3)	4 (21.1)	0.213
Clinical pregnancy, *n* (%)	15 (75.0)	15 (60.0)	0.289

Data are presented as mean ± standard deviation or number (percentage)

*BMI* body mass index, *AMH* anti-müllerian hormone

**Table 5:** Association between vaccination and pregnancy outcomes on crude and adjusted analysis

	Crude OR (95% CI)	Adjusted OR (95% CI)^*^	Adjusted *P*-value^*^
Biochemical pregnancy	1.26 (0.30–5.28)	1.34 (0.23–7.84)	0.749
Biochemical pregnancy loss	0.25 (0.03–2.51)	0.46 (0.05–4.71)	0.512
Clinical pregnancy	2.00 (0.55–7.27)	6.21 (0.76–50.88)	0.089

*OR* odds ratio, *CI* confidence interval

^*^Adjusted for female age and body mass index, male vaccination status, infertility type, duration and diseases, anti-müllerian hormone, antral follicle count, endometrial thickness, embryo stage, expansion grade, inner cell mass grade, and trophectoderm grade

## Discussion

The results of our retrospective cohort study revealed that inactivated SARS-CoV-2 vaccination in females had no detrimental impact on embryo ploidy during IVF treatment. Early pregnancy outcomes were also unaltered in subsequent euploid blastocyst transfer cycles, which provides further reassurance for vaccinated women who are planning to conceive.

Ploidy status of embryos has been recognized as a principal contributing factor in implantation failure and spontaneous abortion [18]. Generally, aneuploidy is caused by meiotic errors during gametogenesis, which generates nullisomic or disomic oocytes or sperms and later leads to abnormal numerical chromosomal constitution in embryos [25]. On the contrary, mosaicism results from mitotic segregation errors in cleaving embryos after fertilization, involving a variety of mechanisms such as anaphase lag, mitotic nondisjunction, inadvertent chromosome demolition, and premature cell division before DNA duplication [26]. In IVF practice, different studies have shown that both ovarian stimulation parameters (e.g., stimulation protocol and treatment duration) and culture conditions (e.g., media type, pH, oxygen, osmolality and temperature) are closely associated with the incidence of aneuploidy and mosaicism, apart from the major influence of maternal age [18, 27–29].

Administration of SARS-CoV-2 vaccine could induce high titers of antigen-specific neutralizing antibodies for CD8 + and Th1 type CD4 + T cells [30]. In addition to serum level changes, anti-SARS-CoV-2 IgG was also detected in the follicular fluid (FF) after vaccination [14, 16], suggesting the local involvement of ovary in immune response. More recently, Castiglione Morelli et al*.* [31] found that vaccinated women had higher FF concentrations of Ala and Pro, while levels of lipids, trimethylamine N-oxide and tumor necrosis factor α were lower compared with non-vaccinated control. Therefore, these immunological, metabolic and inflammatory alterations may interfere with the process of oocyte growth, development and maturation during stimulation, consequently resulting in abnormal fertilization and embryo ploidy [32, 33]. In this context, the present study was conducted based on a cohort of 133 patients undergoing PGT-A cycles. Our results clearly demonstrated that female inactivated SARS-CoV-2 vaccination did not influence the number or proportion of euploid, aneuploid and mosaic blastocysts, implying a limited clinical significance of these biological changes.

To the best of our knowledge, only one prior study has explored the impact of mRNA COVID-19 vaccine (Pfizer or Moderna) on embryo ploidy [17]. In the retrospective cohort by Aharon et al*.* [17], 222 fully vaccinated patients were compared with 983 unvaccinated patients, among whom the proportion of embryo biopsy for PGT-A was 79.7% and 78.6%, respectively. On crude analysis, the euploidy rate was significantly higher in vaccinated women (48.8% [95% CI 44.1–53.6] vs. 42.5% [95% CI 40.2–44.9]; *P* = 0.02), while no significant association was found after controlling for age, BMI, AMH, gravidity, parity, and stimulation protocol (β = 0.05 ± 0.03; *P* = 0.08). Differently, patients in our cohort had a lower proportion of euploid embryos (28.6% [135/472]), which could be attributed to the specific application indications required for PGT-A such as advanced female age (48.1% [64/133]). Nonetheless, the results provide consistent evidence that inactivated SARS-CoV-2 vaccines similarly do not affect embryo ploidy in ovarian stimulation cycles.

Several studies to date have also assessed the IVF pregnancy outcomes following COVID-19 vaccination, with no significant differences observed in biochemical, clinical or ongoing pregnancy rates [15–17, 34–36]. By analyzing euploid blastocyst transfer cycles, our cohort offers a unique opportunity to investigate independently the possible detrimental effect of vaccine-induced autoimmunity on embryo implantation and pregnancy establishment. Indeed, vaccination might promote the production of antiphospholipid antibodies, such as anti-cardiolipin, anti-b2 glycoprotein I and anti-phosphatidylserine/prothrombin [37, 38]. More seriously, it could even trigger the development of immune thrombosis and thrombocytopenia in some cases [39]. These immunological changes have been shown to be closely related to female infertility, implantation failure as well as pregnancy loss [40]. Contrarily, our data showed no association of inactivated SARS-CoV-2 vaccines with early pregnancy outcomes after IVF, suggesting that the hypothesized autoimmune induction may be minimal and nonsignificant.

This study has some limitations that should be acknowledged. Firstly, potential bias and confounding factors are inherent of retrospective cohort studies. In this regard, vaccine data were collected via ascertained immunization records to reduce recall bias by patients, and multivariate linear or logistic analyses were performed for adjusted estimates. Nonetheless, some possible confounders may still be missing such as serum and FF concentrations of SARS-CoV-2 neutralizing antibodies. Indeed, a recent study by Herrero et al*.* [41] suggested that the number of retrieved oocytes and mature oocytes decreased significantly with higher titers of SARS-CoV-2 IgG antibodies in recovered COVID-19 patients. While further studies focusing on patients receiving mRNA vaccines denied this relationship [14, 16], it remains for validation whether the conclusion applies to inactivated vaccines as well. In addition, the history of SARS-CoV-2 infection was determined by patient self-report rather than seropositivity measurement. Therefore, the bias caused by withholding information from patients could also present a confounding risk since those unvaccinated but priorly infected patients should be deemed immunized as those vaccinated [42]. Secondly, our study was conducted in a single center with small sample size, especially in terms of pregnancy outcomes. Although this guarantees the uniformity of clinical practice and laboratory techniques between the two groups, the robustness and generalizability of our conclusion are also limited. Based on a mean euploidy rate of 23% (± 25%) in our center, a sample size of 196 PGT-A patients was required to detect a mean difference of 10% in the primary outcome of euploidy rate with 80% power and type I error of 0.05. With regards to clinical pregnancy rate *per* euploid FET cycle, at least 164 patients should be included for each group to detect an absolute difference of 15%, based on a 67% clinical pregnancy rate from our prior data. Therefore, the current cohort with 133 patients could be possibly underpowered to detect statistical differences in outcomes. Besides, we were unable to carry out further reliable subgroup analyses according to vaccine manufacturer and time interval from vaccination to cycle initiation. Thirdly, the ploidy status of each embryo was determined by PGT-A of trophectoderm biopsy. While the sensitivity and specificity of NGS method approach 100% for aneuploid and euploid embryos [43, 44], there remains a possibility of classification error with false-positive or false-negative results. Finally, owing to the recent application of SARS-CoV-2 vaccines, data on live birth rates as well as obstetrical and neonatal outcomes are currently unavailable, which deserve continuous monitoring in future studies.

## Conclusions

In summary, our study demonstrated for the first time that administration of inactivated SARS-CoV-2 vaccines was not associated with adverse effects on embryo ploidy status or early pregnancy outcomes after IVF. These findings contribute to the ever-increasing evidence regarding the reproductive safety of COVID-19 vaccination in women who are planning to conceive. Further prospective cohort studies with larger sample size and longer follow-up duration are warranted to confirm the conclusion.

## Data Availability

The datasets used and/or analyzed during the current study are available from the corresponding author on reasonable request.

## Abbreviations

AFC: Antral follicle count
AMH: Anti-müllerian hormone
BMI: Body mass index
CI: Confidence interval
COVID-19: Coronavirus disease 19
DNA: Deoxyribonucleic acid
EMT: Endometrial thickness
FET: Frozen-thawed embryo transfer
FF: Follicular fluid
GnRH: Gonadotropin-releasing hormone
hCG: Human chorionic gonadotrophin
IVF: In vitro fertilization
MII: Metaphase II
mRNA: Messenger ribonucleic acid
NGS: Next-generation sequencing
OR: Odds ratio;
PGT-A: Preimplantation genetic testing for aneuploidy
PN: Pronuclei
SARS-CoV-2: Severe acute respiratory syndrome coronavirus
